# Regularized Laplacian determinants of self-similar fractals

**DOI:** 10.1007/s11005-017-1027-y

**Published:** 2017-11-22

**Authors:** Joe P. Chen, Alexander Teplyaev, Konstantinos Tsougkas

**Affiliations:** 1 Colgate Universty, Hamilton, NY 13346 USA; 2 University of Connecticut, Storrs, CT 06269 USA; 3 Uppsala University, 751 05 Uppsala, Sweden

**Keywords:** Regularized determinant, Fractal Laplacian, Spectral zeta functions, Sierpiński gasket, 28A80, 05C30, 58C40, 58J52, 81Q10

## Abstract

We study the spectral zeta functions of the Laplacian on fractal sets which are locally self-similar fractafolds, in the sense of Strichartz. These functions are known to meromorphically extend to the entire complex plane, and the locations of their poles, sometimes referred to as complex dimensions, are of special interest. We give examples of locally self-similar sets such that their complex dimensions are not on the imaginary axis, which allows us to interpret their Laplacian determinant as the regularized product of their eigenvalues. We then investigate a connection between the logarithm of the determinant of the discrete graph Laplacian and the regularized one.

## Introduction

If we have a differential Laplace operator ${\mathcal {L}}$ with discrete spectrum, we can define its spectral zeta function as

$$\begin{aligned} \zeta _{{\mathcal {L}}}(s)=\text {Tr}\left\{ \frac{1}{{\mathcal {L}}^s}\right\} =\sum _n \frac{1}{\lambda _n^s}, \end{aligned}$$

where the zero eigenvalue is excluded and eigenvalues are added according to their multiplicities. Equivalently, if we have the heat kernel trace $K(t)=\sum _n e^{-\lambda _n t}$, we can define the spectral zeta function as the Mellin transform of the heat kernel trace minus one to remove the eigenvalue zero, namely:

$$\begin{aligned} \zeta _{{\mathcal {L}}}(s)=\frac{1}{\varGamma (s)}\int _0^{\infty } (K(t)-1) t^{s-1} \hbox {d}t. \end{aligned}$$

Now we can write formally

$$\begin{aligned} \det {\mathcal {L}}=\prod _{i=1}^{\infty } \lambda _i \end{aligned}$$

to be the product of its nonzero eigenvalues, and we make the convention for the rest of this paper that the zero eigenvalue will always be excluded from any determinant. Of course, in the cases that we will consider the eigenvalues diverge to infinity, so this product exists only in a formal sense. We are interested, however, in assigning some meaning to it, which we can do by the following formal observations

$$\begin{aligned} \zeta _{{\mathcal {L}}}'(s)=\left( \sum _{k=1}^{\infty } \frac{1}{\lambda _i^s}\right) '=- \sum _{k=1}^{\infty } \frac{1}{\lambda _i^s} \log {\lambda _i}. \end{aligned}$$

Evaluating at $s=0$ we get

$$\begin{aligned} \zeta '_{{\mathcal {L}}}(0)=-\sum _{k=1}^{\infty } \log {\lambda _i}=\log {\prod _{i=1}^{\infty } \lambda _i}=-\log {\det {\mathcal {L}}}, \end{aligned}$$

so we can define the determinant of the operator ${\mathcal {L}}$ to be $\det {\mathcal {L}}=e^{-\zeta '_{{\mathcal {L}}}(0)}$.

The spectrum of the Laplace operator on fractals has been the focus of considerable work, see e.g., [9, 10, 19, 22, 29, 30, 32, 33, 35]. Given a post-critically finite (p.c.f.) self-similar set (see [21] for the definition of p.c.f.), one can compute its spectral dimension $d_\mathrm{s}$ and walk dimensions $d_\mathrm{w}$, and these dimensions are connected via the Einstein relation $d_\mathrm{s}=2\frac{d_\mathrm{f}}{d_\mathrm{w}}$ where $d_\mathrm{f}$ is the Hausdorff dimension. In [12, 31, 37, 38], the spectral zeta functions have been studied, and while they are defined initially only for $s>\frac{d_\mathrm{s}}{2}$, they are shown to meromorphically extend to the entire complex plane. Their poles, also called *complex dimensions* [24], are studied in [31] and it is proven that for a large class of p.c.f. fractals with symmetries, that the poles can only be on the imaginary axis or on the axis where ${Re}(s)=\frac{d_\mathrm{s}}{2}$.

Our long-term motivation comes from quantum physics, in particular such recent papers as [1–5, 14, 15, 25, 28, 36] and more classical works [16, 17, 20, 23]. Our immediate mathematical motivation is twofold. On the one hand, it comes from the following statement found in [12] and [13]:

“If there were no poles on the imaginary axis, then $e^{-\zeta '_{\varDelta }(0)}$ would be the regularized product of eigenvalues or the Fredholm determinant of $\varDelta$.”

On the other hand, in [11] a connection between the determinant of the discrete Laplacians and the regularized determinant has been made in the setting of the discrete Euclidean torus. Specifically, let $N=(n_1(u), \ldots n_d(u))$ denote a *d*-tuple of positive integers parametrized by $u \in {\mathbb {Z}}$, such that for each *j*, we have $\frac{n_j(u)}{u} \rightarrow a_j$ as $u \rightarrow \infty$. One then defines the *d*-dimensional discrete torus as the product space

$$\begin{aligned} DT_{N(u)}=\prod _{i=1}^d n_j(u){\mathbb {Z}} / {\mathbb {Z}}. \end{aligned}$$

If *A* is the diagonal matrix with entries $a_j$ and $V(a)=a_1\cdots a_d$, the authors of [11] established the formula

1.1 $$\begin{aligned} \log {\det \varDelta _{DT_{N(u)}}}=V(N(u)) {\mathcal {I}}_d(0)+\log {u^2}+\log {\det \varDelta _{RT,A}}+o(1) \text { as } u\rightarrow \infty , \end{aligned}$$

where *RT*, *A* is the real torus $A {\mathbb {Z}}^d / {\mathbb {R}}^d$, and ${\mathcal {I}}$ is a specific special function. A variation of this result was also studied in [40].

The goal of this paper is to give examples of fractals whose spectral zeta functions have no poles on the imaginary axis, which then allows us to define the corresponding Laplacian determinant, interpreted as the regularized product of the Laplace eigenvalues. This result can be stated as a regularized limit and has been proven again with a different methodology in [40]. More specifically, if $f \in C^{\infty }({\mathbb {R}}^{+},{\mathbb {C}})$ and is of the form

$$\begin{aligned} f(x)=\sum _{j=1}^{N-1}\sum _{k=0}^{M_j} a_{jk} x^{a_j}\log ^k{x}+\sum _{k=0}^{M_0}a_{0k} \log ^k{x}+o\left( x^{a_N}\log ^{M_N}{x}\right) \end{aligned}$$

for some $N \in {\mathbb {N}}$, $(a_j) \subset {\mathbb {C}}$ such that $({Re}(a_j))$ is monotonically decreasing and ${Re}(a_N)<0$ then we define the regularized limit of *f* as $\widetilde{\lim _{x \rightarrow \infty }} f(x)=a_{00}.$ Then as in [40] we can restate the above result as

$$\begin{aligned} \widetilde{\lim _{n \rightarrow \infty }} \log \det \varDelta _n=\log \det \varDelta . \end{aligned}$$

Motivated by the above-mentioned connection between a classical determinant and a zeta regularized determinant, we investigate a similar relation on some fractal examples. In this paper, we study three concrete examples: the diamond fractal, the $$N-1$$-dimensional double Sierpiński gaskets ($$SG^N)$$, and the double *pq*-model on the unit interval. All three examples satisfy spectral decimation, which leads to closed-form expressions for the spectral zeta functions and the Laplacian determinants. However, only for the double Sierpiński gaskets and the double *pq*-model do we have exact analogs of (1.1). Details will be described in subsequent sections, after a review of basic notions from analysis on fractals and graph theory.

## Notions of analysis on fractals and graph theory

The fractals we will study are self-similar sets defined in the following way. Given a compact connected metric space (*X*, *d*), and injective contractions $F_i: X \rightarrow X$, $i \in \{1,2,\ldots , m\}$, there exists a unique non-empty compact subset *K* of *X* that satisfies

$$\begin{aligned} K=\bigcup _{i=1}^m F_i(K). \end{aligned}$$

This will be our self-similar set. A fixed point $p_1$ of one of the maps $F_i$ for some $1\le i\le m$ is called an essential fixed point if there exists another fixed point $p_{2}$ such that $F_{j} (p_{1}) = F_{k} (p_{2})$ for some $(1 \le j = k \le m)$. Associated with *K* is a sequence of approximating graphs $\{G_n : n\ge 0\}$, defined as follows. Let $V_0$ be the set consisting of the essential fixed points of the maps $F_i$ and $G_{0}$ be the complete graph on $V_{0}$. For $n\ge 1$, we define inductively

$$\begin{aligned} V_n:= \bigcup _{i=1}^m F_i(V_{n-1}), \end{aligned}$$

and declare $x,y\in V_n$ to be connected by an edge in $G_n$ (denoted $x\underset{n}{\sim } y$) if $F_i^{-1}(x)$ and $F_i^{-1}(y)$ are connected by an edge in $G_{n-1}$ for some $1\le i\le m$. We define the Dirichlet form on $G_n$ in the usual way

$$\begin{aligned} {\mathcal {E}}_m(u,v)= \sum _{x\underset{m}{\sim } y} \left( u(x)-u(y) \right) \left( v(x)-v(y) \right) , \quad u,v: V_m\rightarrow {\mathbb {R}}. \end{aligned}$$

In many fractal examples, it is possible to show that $\displaystyle \lim \nolimits _{n\rightarrow \infty } r^{-n} {\mathcal {E}}_n(u,v)$ exists, where $r>0$ is a renormalization constant. We denote this limit by ${\mathcal {E}}(u,v)$ and write ${\mathcal {E}}(u)$ to stand for ${\mathcal {E}}(u,u)$. For example, the standard two-dimensonal

Sierpiński gasket $SG_2$ satisfies this property with $r=\frac{3}{5}$. Every function of finite energy is continuous. In fact $\hbox {dom}{\mathcal {E}}$, the space of functions of finite energy is a dense subspace of the space of continuous functions on *K*. Given the energy, we can define the Laplacian which is the main focus of our study. For $u \in \hbox {dom}{\mathcal {E}}$ we say $u \in \hbox {dom}\varDelta _{\mu }$ and $\varDelta _{\mu }u=f$ if

$$\begin{aligned} {\mathcal {E}}(u,v)=- \int _K {fv} \mathrm {d\mu } \text { for all } v\in \hbox {dom}_0 {\mathcal {E}} \end{aligned}$$

where $\hbox {dom}_0 {\mathcal {E}}$ denotes the subset of $\hbox {dom} {\mathcal {E}}$ such that the functions also vanish on the boundary. We will use the convention from now on $-\varDelta _{\mu }={\mathcal {L}}$, and we always assume that $\mu$ will be the standard self-similar measure so it will be omitted from the notation.

### Spectral decimation and zeta functions

The Laplace operator ${\mathcal {L}}$ with Neumann or Dirichlet conditions is a nonnegative self-adjoint operator with compact resolvent. Its spectrum consists of discrete eigenvalues such that

$$\begin{aligned} 0 \leqslant \lambda _1 < \lambda _2 \leqslant \lambda _3 \leqslant \cdots \end{aligned}$$

with $\lambda _n \rightarrow \infty$, and thus, we can define its spectral zeta function as in the introduction. The key technique to studying the spectrum of the Laplacian on fractals is spectral decimation. Essentially, spectral decimation allows us to recursively obtain the eigenvalues of a given graph level approximation from knowledge of the previous graph approximation. In the end, taking a limit gives us the spectrum of the Laplace operator on the self-similar set. More rigorously, we say that we have spectral decimation if all eigenvalues of ${\mathcal {L}}$ are of the form

$$\begin{aligned} - \lambda ^m \lim _{n\rightarrow \infty } \lambda ^n R^{(-n)}(w) \end{aligned}$$

for $w \in A$, where *A* is a finite set and *R* is a rational function. For the limit to exist the elements of the preimages $R^{-(n)}(w)$ must be chosen appropriately. The value *m* stands for the so-called generation of birth of the eigenvalue *w* and is independent of *n*; more information may be found at [12, 19, 34, 38]. In many cases, this rational function turns out to be a polynomial. The quantity $\lambda$ is also known as the time scaling factor.

Now, let $R(z)=a_dx^d+\dots +\lambda x$ be a polynomial with real coefficients and $d\ge 2$ which satisfies $R(0)=0$ and $R'(0)= \lambda > 1$. We denote as $\varPhi$ the entire function which is a solution of the functional equation

$$\begin{aligned} \varPhi (\lambda z)=R(\varPhi (z)) \text { with } \varPhi (0)=0, \varPhi ' (0)=1. \end{aligned}$$

We also define the so-called polynomial zeta functions

$$\begin{aligned} \zeta _{\varPhi ,w}(s)= \sum \limits _{\begin{array}{c} \varPhi (-\mu )=w \\ \mu >0 \end{array}} \mu ^{-s} \end{aligned}$$

or equivalently as

$$\begin{aligned} \zeta _{\varPhi ,w}(s)= \lim _{n \rightarrow \infty } \sum _{z \in R^{-n}(w)} (\lambda ^n z)^{-s}. \end{aligned}$$

These zeta functions have been studied in [12, 38] and were used in the meromorphic extension of the spectral zeta functions. We know the following facts about them. For $w<0$, we have that

$$\begin{aligned} \zeta _{\varPhi ,w}(0)=0 \quad \text { and } \quad \zeta '_{\varPhi ,w}(0)=\frac{\log {a_d}}{d-1}+\log {(-w)} \end{aligned}$$

and for $$w=0$$

$$\begin{aligned} \zeta _{\varPhi ,0}(0)=1 \quad \text { and } \quad \zeta '_{\varPhi ,0}(0)=\frac{\log {a_d}}{d-1}. \end{aligned}$$

Moreover, they can be meromorphically extended to the entire complex plane and have no poles on the imaginary axis. More specifically, all the poles are simple and are located on the imaginary line where ${Re}(s)=\frac{\log {d}}{\log {\lambda }}$. For further information we refer the reader to [12, 38].

### Counting spanning trees in fractal graphs

In the graph theoretic setting, the determinant of the discrete Laplacian is widely studied, as it is related to the enumeration of spanning trees via Kirchhoff’s Matrix-Tree theorem. To be concrete, we define the *combinatorial graph Laplacian* of a graph $G_n$ as $\varDelta _n=D-A$, and the *probabilistic graph Laplacian* as ${\mathcal {L}}_n=I-D^{-1}A$, where *D* is the diagonal degree matrix and *A* is the adjacency matrix. Then the number of spanning trees in $G_n$ may be expressed in either of two ways:

$$\begin{aligned} \tau (G_n)=\frac{\det \varDelta _n}{|V_n|} \quad \text { or }\quad \tau (G_n)=\left( \frac{\prod _i d_i}{\sum _i d_i}\right) \det {\mathcal {L}}_n, \end{aligned}$$

where $d_i$ are the vertex degrees. One can further introduce the *asymptotic complexity constant* of $(G_n)$, studied in [26],

$$\begin{aligned} c=\lim _{n \rightarrow \infty } \frac{\log {\tau (G_n)}}{|V_n|} \end{aligned}$$

provided that the limit exists.

For fractal graphs admitting spectral decimation, the determinant of the graph Laplacians, as well as the asymptotic complexity constant, has been evaluated in [6]. The key insight is that one can split the eigenvalues into two disjoint finite sets *A* and *B*. If the rational function associated with spectral decimation is of the form $R(z)=\frac{P(z)}{Q(z)}$ with degree *d*, and $P_d$ is the leading coefficient of *P*, then

2.1 $$\begin{aligned} {} \det {\mathcal {L}}_n=\left( \prod _{\alpha \in A}\alpha ^{\alpha _n}\right) \left[ \prod _{\beta \in B}\left( \beta ^{\sum _ {k=0}^{n}{\beta _n^k}}\left( \frac{-Q(0)}{P_d}\right) ^{\sum _{k=0}^n\beta _n^k\left( \frac{d^k-1}{d-1}\right) }\right) \right] . \end{aligned}$$

where $\alpha _n=\hbox {mult}_n(\alpha )$ and $\beta _n^k=\hbox {mult}_nR^{-k}(\beta )$, where $\hbox {mult}_n(\lambda )$ denotes the multiplicity of the eigenvalue $\lambda$ of ${\mathcal {L}}_n$. We refer the reader to [6, 7] and [27] for details.

## Zeta function of the diamond fractal

**Fig. 1:**
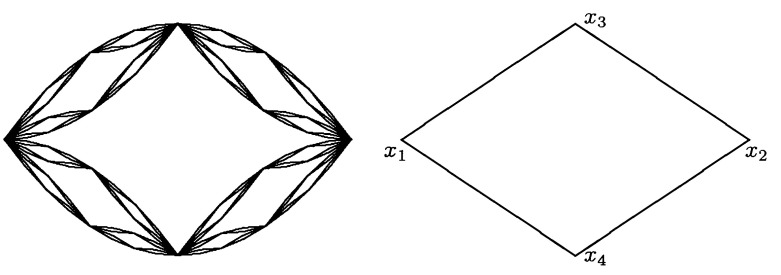
Diamond fractal and its $G_1$ approximating graph

The diamond fractal has been recently studied due to its connections related to physics. In this section, we show that it is a fractal with spectral zeta function such that it has no poles on the imaginary axis. Moreover, we establish a connection between the discrete and continuous determinants of the Laplacian bearing some resemblance to [11]. We depict the diamond fractal and its first graph approximation in (Fig. 1).

### Proposition 3.1

The spectral zeta function of the diamond fractal factorizes as follows

$$\begin{aligned} \zeta _{{\mathcal {L}}}(s)=\frac{4^s(4^s-1)}{3}\left( \frac{4}{4^s-4}+\frac{2}{4^s-1}\right) \zeta _{\varPhi ,0}(s) \end{aligned}$$

and thus has no poles on the imaginary axis. Its regularized determinant is

$$\begin{aligned} \det {\mathcal {L}}=2^{-\frac{10}{9}} \end{aligned}$$

### Proof

For the diamond fractal spectral decimation has been done in [7] and it was obtained that

$$\begin{aligned} R(z)=2z(2+z) \text { and } \lambda = R'(0)=4. \end{aligned}$$

Then $\varPhi$ satisfies the functional equation $\varPhi (\lambda z)=R(\varPhi (z))$ and thus we have that $\varPhi (4z)=2\varPhi (z)(2+\varPhi (z))$. This allows us to say that

$$\begin{aligned} \varPhi (z)=-1 \Leftrightarrow \varPhi (4z)=-2 \end{aligned}$$

and

$$\begin{aligned} \varPhi (z)=-2 \Leftrightarrow \varPhi (4z)=0 \quad \text {and}\quad \varPhi (z) \ne 0. \end{aligned}$$

We have that every eigenvalue of ${\mathcal {L}}$ is of the form $-4^m \lim \nolimits _{n\rightarrow \infty } 4^nR^{-n}(-1)$ and $\hbox {mult}_n(1)=\frac{4^n+2}{3}$. Thus we get that

$$\begin{aligned} \zeta _{{\mathcal {L}}}(s)= \sum _{n=1}^{\infty } \left( \frac{4^n+2}{3}\right) 4^{-ns} \zeta _{\varPhi ,-1}(s)=\frac{1}{3}\left( \frac{4}{4^s-4}+\frac{2}{4^s-1}\right) \zeta _{\varPhi ,-1}(s). \end{aligned}$$

We will show that in fact we have no poles on the imaginary axis because of cancellations from the solutions of $4^s=1$. By using the observations above, we obtain that

$$\begin{aligned} \begin{aligned} \zeta _{\varPhi ,-1}(s)=&\sum \limits _{\begin{array}{c} \varPhi (-\mu )=-1 \\ \mu>0 \end{array}} \mu ^{-s}=\sum \limits _{\begin{array}{c} \varPhi (-4\mu )=-2 \\ \mu>0 \end{array}} \mu ^{-s}= 4^s \sum \limits _{\begin{array}{c} \varPhi (-4\mu )=-2 \\ \mu >0 \end{array}} (4\mu )^{-s}= 4^s \zeta _{\varPhi ,-2}(s) \\ \end{aligned} \end{aligned}$$

and

$$\begin{aligned} \begin{aligned} \zeta _{\varPhi ,-2}(s)&= \sum \limits _{\begin{array}{c} \varPhi (-\mu )=-2 \\ \mu>0 \end{array}} \mu ^{-s}=\sum \limits _{\begin{array}{c} \varPhi (-4\mu )=0 \\ \varPhi (-\mu ) \ne 0 \\ \mu>0 \end{array}} \mu ^{-s}= \sum \limits _{\begin{array}{c} \varPhi (-4\mu )=0 \\ \mu>0 \end{array}} \mu ^{-s}-\sum \limits _{\begin{array}{c} \varPhi (-\mu )=0 \\ \mu>0 \end{array}} \mu ^{-s}\\&=4^s\sum \limits _{\begin{array}{c} \varPhi (-4\mu )=0 \\ \mu>0 \end{array}} (4\mu )^{-s}-\sum \limits _{\begin{array}{c} \varPhi (-\mu )=0 \\ \mu >0 \end{array}} \mu ^{-s}= (4^s-1) \zeta _{\varPhi ,0}(s) \end{aligned} \end{aligned}$$

From this it follows that

$$\begin{aligned} \zeta _{{\mathcal {L}}}(s)=\frac{4^s(4^s-1)}{3}\left( \frac{4}{4^s-4}+\frac{2}{4^s-1}\right) \zeta _{\varPhi ,0}(s) \end{aligned}$$

which proves that there are no poles on the imaginary axis.

Now by differentiating and using the fact that $\zeta _{\varPhi ,0}(0)=1$ and that $\zeta '_{\varPhi , 0}(0)=\frac{\log {a_d}}{d-1}=\log {2}$ we obtain $\zeta '_{{\mathcal {L}}}(0)=\frac{10}{9}\log {2}$. But due to the discussion in the introduction this essentially means that the absence of poles on the imaginary axis allows us to interpret this as the regularized product of the eigenvalues and thus

$$\begin{aligned} \det {\mathcal {L}}=e^{-\zeta '_{{\mathcal {L}}}(0)}=2^{-\frac{10}{9}}. \end{aligned}$$

$$\square $$

### Remark 3.1

At first glance, the complex dimensions would be located at the positions such that $4^s=4$ which are $s=1+\frac{ik\pi }{\log {2}}$ and at ${Re}(s)=\frac{\log {2}}{\log {4}}=\frac{1}{2}$ due to the poles of the polynomial zeta functions. However, it was shown in [31] that the complex dimensions can only be on the imaginary axis, which we have proven is not the case, and at ${Re}(s)=\frac{d_\mathrm{s}}{2}=1$ and thus we can deduce that all the poles of the polynomial zeta functions must be canceled by the zeros of the geometric part which we observe that is indeed the case for ${Re}(s)=\frac{1}{2}$.

### Remark 3.2

The value $\log {2}$ can also be interpreted as the tree entropy or the asymptotic complexity constant of the sequence of the fractal graphs approximating the diamond fractal. Thus $\log {\det {\mathcal {L}}}=-\frac{10}{9}c$.

For the diamond fractal, it has been calculated in [6] that

$$\begin{aligned} \det {\mathcal {L}}_n=2^{-\frac{1}{9}(2\cdot 4^n-6n-11)}. \end{aligned}$$

Then using the fact that $\log {\det {\mathcal {L}}}=-\frac{10}{9}\log {2}$ we conclude that

$$\begin{aligned} \log {\det {\mathcal {L}}_n}=\frac{1}{10}(2\cdot 4^n -6n-11) \log {\det {\mathcal {L}}} \end{aligned}$$

We can see that the regularized determinant does not appear as an exact constant as in [11] despite the fact that there are no poles on the imaginary axis. The resemblance that appears here must be attributed to numerical coincidence.

There is also a quantum graph interpretation of the diamond fractal with spectral zeta function

$$\begin{aligned} \zeta _D(s)=\frac{\zeta _R(2s)}{\pi ^{2s}}l^{d_\mathrm{f}-1}\left( \frac{1-l^{1-d_\mathrm{ws}}}{1-l^{d_\mathrm{f}-d_\mathrm{ws}}} \right) \end{aligned}$$

where $\zeta _R$ is the Riemann zeta function, $\lambda$ is a side length constant and $d_\mathrm{w}$ and $d_\mathrm{f}$ are correspondingly the walk and Hausdorff dimensions.

## Zeta function of double Sierpiński gaskets

The spectral zeta functions for Dirichlet and Neumann boundary conditions for the standard self-similar Laplacian on the standard two-dimensional Sierpiński gasket have been calculated in [12, 38] as follows:

$$\begin{aligned} \begin{aligned} \zeta _{{\mathcal {L}}}^D(s)&=5^{-s}\zeta _{\varPhi ,-2}(s)+\left( \frac{3}{2(5^s-3)}-\frac{3}{2(5^s-1)} \right) 5^{-s}\zeta _{\varPhi ,-3}(s) \\&\quad +\left( \frac{1}{2(5^s-3)}+\frac{3}{2(5^s-1)} \right) \zeta _{\varPhi ,-5}(s) \end{aligned} \end{aligned}$$

and

$$\begin{aligned} \zeta _{{\mathcal {L}}}^N(s)=\left( \frac{1}{2(5^s-3)}+\frac{3}{2(5^s-1)} \right) \zeta _{\varPhi ,-3}(s) +\left( \frac{3\cdot 5^{-s} }{2(5^s-3)}-\frac{5^{-s}}{2(5^s-1)} \right) \zeta _{\varPhi ,-5}(s). \end{aligned}$$

Notice that the poles on the imaginary axis appear to be at the points such that $5^s=1$. However, some are canceled out by the observation in [12] that $\zeta _{\varPhi , -5}(s)=(5^s-1) \zeta _{\varPhi ,0}(s)$. Unfortunately, a similar argument cannot work for $\zeta _{\varPhi ,-3}(s)$ and numerical calculations by the authors in [12] indicate that we indeed have poles at $5^s=1$.

**Fig. 2:**
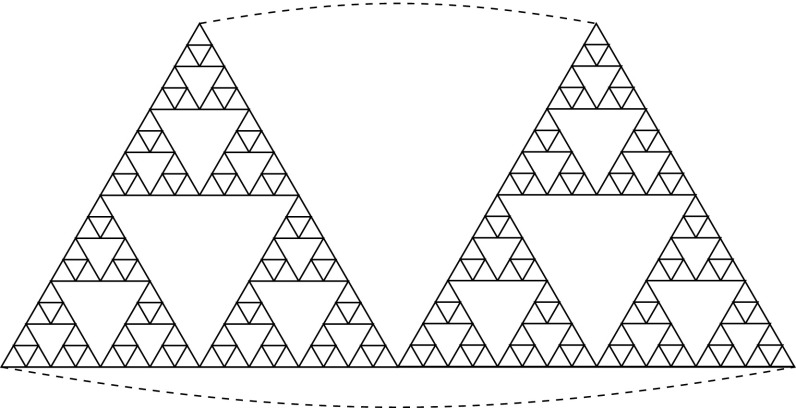
Approximating graph $G_4$ of the double Sierpiński gasket. Points connected by a dashed line are identified

In [32] the double Sierpiński gasket was defined. Essentially, it is the fractal created by taking two copies of the regular Sierpiński gasket and gluing them at the boundary. Then it becomes a fractal without boundary and its graph approximations are 4-regular graphs (Fig. 2).

We can also consider higher-dimensional analogues of the Sierpiński gasket. Denote $SG^N$ to be the $N-1$-dimensional Sierpiński gaskets as in [19]. The time scaling factor is then $\lambda =N+2$. The Dirichlet spectral zeta function is evaluated in [12]

$$\begin{aligned} \begin{aligned} \zeta _{{\mathcal {L}}}^D(s)&=\lambda ^{-s}\zeta _{\varPhi ,-2}(s)+\left( \frac{(N-1)^2-1}{2(\lambda ^{s}-N)}-\frac{N}{2(\lambda ^{s}-1)} \right) \lambda ^{-s} \zeta _{\varPhi , -N}(s)\\&\quad +\left( \frac{N-2}{2(\lambda ^{s}-N)}+\frac{N}{2(\lambda ^{s}-1)} \right) \zeta _{\varPhi ,-(N+2)}(s) \end{aligned} \end{aligned}$$

and by using explicit knowledge of the Neumann spectrum from [7, 19], we can also compute the Neumann spectral zeta function to be

$$\begin{aligned} \begin{aligned} \zeta _{{\mathcal {L}}}^N(s)&=\left( \frac{N-2}{2(\lambda ^s-N)}+\frac{N}{2(\lambda ^s-1)}\right) \zeta _{\varPhi , -N}(s)\\&\quad +\left( \frac{N(N-2)}{2(\lambda ^s-N)}-\frac{N-2}{2(\lambda ^s-1)}\right) \lambda ^{-s}\zeta _{\varPhi ,-(N+2)}(s). \end{aligned} \end{aligned}$$

We can now create the double $SG^N$ by taking two copies of $SG^N$ and gluing them together at the respective boundary points, making the appropriate *N* identifications. Then it becomes a fractal without boundary and the spectrum of the Laplace operator is the union of the Dirichlet and Neumann spectra with added multiplicities.

### Proposition 4.1

The spectral zeta function of the double $N-1$-dimensional Sierpiński gasket has no poles on the imaginary axis. Its regularized determinant is

$$\begin{aligned} \det {\mathcal {L}}=\frac{(N+2)^{\frac{N-2}{N-1}}}{2N^{\frac{1}{N-1}}}. \end{aligned}$$

### Proof

The spectral zeta function for the double $SG^N$ is the sum of the Dirichlet and Neumann spectral zeta functions of the single gaskets and thus becomes

$$\begin{aligned} \zeta _{{\mathcal {L}}} (s) = \lambda ^{-s} \zeta _{\varPhi ,-2}(s)+ \frac{N(\lambda ^s-1)-\lambda ^s}{\lambda ^s(\lambda ^s-N)}\zeta _{\varPhi ,-N}(s) +\frac{(N-1)(\lambda ^s-2)}{(\lambda ^s-1)(\lambda ^s-N)}\zeta _{\varPhi ,-(N+2)}(s). \end{aligned}$$

But since as in [12] we have that $\zeta _{\varPhi ,-(N+2)}(s)=(\lambda ^s-1)\zeta _{\varPhi ,0}(s)$ we see that we don’t have any poles on the imaginary axis which allows us to have the interpretation of a regularized determinant. By differentiating the formula above and taking into account that the spectral decimation function is $R(z)=z(N+2+z)$ with $d=2$, $a_d=1$ and also that

$$\begin{aligned} \begin{aligned}&\zeta _{\varPhi ,0}(0)=1 \quad \text { and } \quad \zeta '_{\varPhi ,0}(0)=0\\&\zeta _{\varPhi ,-N}(0)=0 \quad \text { and } \quad \zeta '_{\varPhi ,-N}(0)=\log {N}\\&\zeta _{\varPhi ,-2}(0)=0 \quad \text { and } \quad \zeta '_{\varPhi ,-2}(0)=\log {2} \end{aligned} \end{aligned}$$

we obtain that

$$\begin{aligned} \zeta '(0)=\log {2}+\frac{1}{N-1}\log {N}-\frac{N-2}{N-1}\log {(N+2)} \end{aligned}$$

and therefore

4.1 $$\begin{aligned} {} \log {\det {\mathcal {L}}}=-\log {2}-\frac{1}{N-1}\log {N}+\frac{N-2}{N-1}\log {(N+2)} \end{aligned}$$

from which the result follows. $\square$

### Remark 4.1

As in the case of the diamond fractal, the zeros of the geometric part cancel all the poles of the polynomial zeta functions, and the only poles that remain are at ${Re}(s)=\frac{d_s}{2}=\frac{\log {N}}{\log {(N+2)}}$.

We establish now a result analogous to [11].

### Corollary 4.1

For the discrete combinatorial graph Laplacian determinant of the double $SG^N$, we have that

$$\begin{aligned} \log {\det \varDelta _n}=c|V_n|+n\log {(N+2)}- \log {\det {\mathcal {L}}}, \end{aligned}$$

where *c* is the asymptotic complexity constant which is

$$\begin{aligned} c=\frac{N-2}{N}\log {2}+\frac{N-2}{N-1}\log {N}+\frac{N-2}{N(N-1)}\log {(N+2)}. \end{aligned}$$

### Proof

By using (2.1), the fact that the spectrum is the union of the Dirichlet and Neumann spectra with added multiplicities and the eigenvalue multiplicities computed at [19, 29] we can evaluate that

$$\begin{aligned} \det \varDelta _n=2^{(N-2)N^n+1}\cdot N^{\frac{(N-2)N^{n+1}+1}{N-1}}\cdot (N+2)^{\frac{(N-2)N^n+n(N-1)-N+2}{N-1}} \end{aligned}$$

By using Kirchhoff’s Matrix-Tree theorem and also the fact that the number of vertices for the double Sierpiński gasket graphs is $|V_n|=N^{n+1}$, we get that the number of spanning trees is

$$\begin{aligned} \tau (G_n)=2^{(N-2)N^n+1}\cdot N^{\frac{(N-2)N^{n+1}-(N-1)(n+1)+1}{N-1}}\cdot (N+2)^{\frac{(N-2)N^n+n(N-1)-N+2}{N-1}} \end{aligned}$$

and therefore the asymptotic complexity constant is

$$\begin{aligned} c=\frac{N-2}{N}\log {2}+\frac{N-2}{N-1}\log {N}+\frac{N-2}{N(N-1)}\log {(N+2)}. \end{aligned}$$

Using (4.1) we obtain the result. $\square$

### Remark 4.2

By this formula, we can see the connection between different “discrete and continuous” determinants. In fact, the asymptotic complexity constant can also be interpreted as a determinant, namely a Fuglede–Kadison determinant. We refer the reader to [18, 26] for more details. We then have a connection between “discrete and continuous” determinants of the form

$$\begin{aligned} \log {\det \varDelta _n}=\log {\text {Det}\varDelta }\,|V_n|+n\log {(N+2)}- \log {\det {\mathcal {L}}}, \end{aligned}$$

where $\text {Det}\varDelta$ is the Fuglede–Kadison determinant.

### Remark 4.3

By using Kirchhoff’s Matrix-Tree theorem and the above calculations, we can also calculate the number of spanning trees for the single $N-1$-dimensional Sierpiński gasket confirming the formula conjectured in [8] and first proven via a different methodology in [39]. The asymptotic complexity constant for the single and double pre-fractal Sierpiński graphs are the same.

## Zeta function of the double pq-model on the unit interval

In [34], the unit interval can be realized as a p.c.f. self-similar set with two contractions. Then the standard self-similar measure is the Lebesgue measure and the fractal Laplacian coincides with the standard $-\frac{\text {d}^2}{\text {d}x^2}$ operator. However, in [38] a different fractal Laplacian on the unit interval has been constructed. Let $0<p<1$ and $q=1-p$. Define contraction factors

$$\begin{aligned} r_1=r_3=\frac{p}{1+p} \quad \text { and } \quad r_2=\frac{q}{1+p} \end{aligned}$$

and measure weights

$$\begin{aligned} m_1=m_3=\frac{q}{1+q} \quad \text { and } \quad m_2=\frac{p}{1+q} \end{aligned}$$

and observe that $m_1+m_2+m_3=r_1+r_2+r_3=1$. We define the contractions $F_i:{\mathbb {R}} \rightarrow {\mathbb {R}}$ for $i=1,2,3$ as $F_i(x)=r_ix+(1-r_i)p_i$ where $p_i$ is $0,\frac{1}{2},1$, respectively, or equivalently the fixed point of $F_i$. Then the unit interval is the self-similar set created by these contractions and as usual $V_n=\bigcup F_i (V_{n-1})$ with the boundary being $V_0=\{0,1\}$. As our self-similar probability measure, we take the unique measure satisfying $\mu =\sum _{j=1}^3 m_j \mu \circ F_j$ and then we have that

$$\begin{aligned} \varDelta _{\mu }(x)=\lim _{n \rightarrow \infty } \left( 1+\frac{2}{pq}\right) ^n \varDelta _n f(x) \end{aligned}$$

where the discrete graph Laplacians are

$$\begin{aligned} \varDelta _nf(x_k)= \left\{ \begin{array}{ll} 2pf(x_{k-1})+2qf(x_{k+1})-2f(x_k) \\ \quad \qquad \qquad \quad \text {or}\\ 2qf(x_{k-1})+2pf(x_{k+1})-2f(x_k) \end{array} \right. \end{aligned}$$

**Fig. 3:**
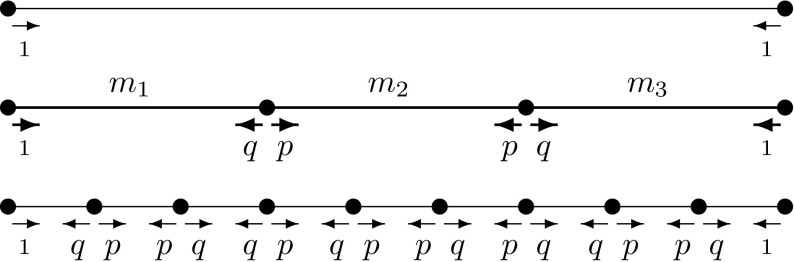
Associated random walk of the *pq*-model

This Laplacian corresponds to a random walk as in Fig. 3.

Spectral decimation has been carried in [38] for the Neumann case with rational function $R_p(z)=\frac{1}{pq}z(\frac{z^2}{4}+\frac{3z}{2}+2+pq)$ and the following is obtained.

### Proposition 5.1

$$\begin{aligned} \sigma (\varDelta _{p,n})=\{0,-4\}\bigcup _{m=0}^{n-1} R_p^{-m}\{-2\pm 2q\} \end{aligned}$$

and if $p \ne \frac{1}{2}$ then $d_\mathrm{s}=\frac{\log {9}}{\log {(1+\frac{2}{pq})}}<1$ and

$$\begin{aligned} \zeta _{{\mathcal {L}}_{\mu }}^N(s)=\frac{1}{\lambda ^{s}-1}(\zeta _{\varPhi , w_1}(s)+\zeta _{\varPhi , w_2}(s)) \end{aligned}$$

for $$\lambda =1+\frac{2}{pq}$$ and $$w_1,w_2=-2 \pm 2q$$.

We can easily see that $R^{-1}(0)=\{0,-2-2p,-2-2q\}$ and $R^{-1}(-4)=\{-4,-2+2p,-2+2q\}$ and thus the spectrum is obtained as follows

This calculation for the spectral zeta function was omitted in [38]. We clarify it here and also fix a typo in the formula. Both eigenvalues $-2+2q$ and $-2-2q$ appear with multiplicity 1 for $m\ge 1$ and thus

$$\begin{aligned} \zeta _{{\mathcal {L}}}(s)= & {} \sum _{m=1}^{\infty }\lambda ^{-ms} \zeta _{\varPhi , -2-2q}(s)+\sum _{m=1}^{\infty }\lambda ^{-ms} \zeta _{\varPhi , -2+2q}(s).\\= & {} \frac{\lambda ^{-s}}{1-\lambda ^{-s}}(\zeta _{\varPhi , -2-2q}(s)+\zeta _{\varPhi , -2+2q}(s)) \end{aligned}$$

Now, we mimic the construction of the double Sierpiński gasket and glue this model with a copy of itself at the two boundary points. Its spectrum is again the union of the Dirichlet and Neumann spectra of the single case and we get the following.

### Proposition 5.2

The spectral zeta function for the double *pq*-model is given by

$$\begin{aligned} \zeta _{{\mathcal {L}}_{\mu }}(s)=\zeta _{\varPhi , 0}(s)+\zeta _{\varPhi ,-4}(s) \end{aligned}$$

and thus it has no poles on the imaginary axis. Its regularized determinant is

$$\begin{aligned} \det {\mathcal {L}}_{\mu }=pq. \end{aligned}$$

Before we give this proof, we must calculate the Dirichlet spectrum for the single *pq*-model. By solving the Dirichlet eigenvalue equation on the first level, we see that the eigenvalues are $-1-p$ and $-1+p$. These eigenvalues are initial and they show up at every level, and we encounter no exceptional eigenvalues by taking their preiterates. This means that the spectrum is of the following form.

Thus while for the Neumann spectrum we have that $\dim \varDelta _n=3\dim \varDelta _{n-1}-2$ for the Dirichlet spectrum we have that $\dim \varDelta _n=3\dim \varDelta _{n-1}+2$. Then the proof of the proposition goes as follows.

### Proof

As in the case of the double Siepriński gasket, it suffices to add the spectral zeta functions of the Neumann and Dirichlet spectrum. Every eigenvalue has multiplicity one and from the calculations above we have that the Dirichlet spectral zeta function is

$$\begin{aligned} \zeta _{{\mathcal {L}}_{\mu }}(s)=\frac{1}{\lambda ^{s}-1}(\zeta _{\varPhi , w_3}(s)+\zeta _{\varPhi , w_4}(s)) \end{aligned}$$

where $w_3,w_4=-2 \pm 2p$. Since $\varPhi (\lambda z)=R(\varPhi (z))$ we can observe that

$$\begin{aligned} \varPhi (z)=-2-2q \Leftrightarrow \varPhi (\lambda z)=0 \text { and } \varPhi (z) \ne 0 \text { and } \varPhi (z) \ne -2-2p \end{aligned}$$

and

$$\begin{aligned} \varPhi (z)=-2+2q \Leftrightarrow \varPhi (\lambda z)=-4 \text { and } \varPhi (z) \ne -4 \text { and } \varPhi (z) \ne -2+2p . \end{aligned}$$

Then, as in the case of the diamond fractal, we have thatand similarly we have that

$$\begin{aligned} \zeta _{\varPhi , -2+2q}(s))=(\lambda ^s-1)\zeta _{\varPhi ,-4}(s)-\zeta _{\varPhi , -2+2p}(s). \end{aligned}$$

The result then is obtained by adding the Dirichlet and Neumann spectral zeta functions and the fact that $\zeta '_{\varPhi ,0}(0)=\frac{\log {\frac{1}{4pq}}}{2}$ and $\zeta '_{\varPhi ,-4}(0)=\frac{\log {\frac{1}{4pq}}}{2}+\log {4}$. $\square$

### Remark 5.1

The location of the poles must necessarily coincide with the location of the poles of the polynomial zeta functions and are thus at ${Re}(s)=\frac{\log {3}}{\log {\lambda }}$.

Then as in [11] we establish that the logarithm of the regularized determinant appears as a constant in the logarithm of the determinant of the discrete graph Laplacians.

### Corollary 5.1

For the double *pq*-model, we have that

5.1 $$\begin{aligned} {} \log {\det \varDelta _n}=|V_n|(\log {2}+\frac{\log {(pq)}}{2})+n\log {\frac{(1-q^2)(1-p^2)}{(pq)^2}}-\log {\det {\mathcal {L}}} \end{aligned}$$

### Proof

First of all, it is easy to calculate that $$|V_n|=2\cdot 3^n$$. Then, we use (2.1) with $$P_d=\frac{1}{4pq}$$, $$ Q_0=1$$ and $$\alpha =-4$$, $$\alpha _n=1$$, $$\beta _1, \beta _2=-2\pm 2q$$, $$\beta _3, \beta _4 =-2 \pm 2p$$ and $$\hbox {mult}_n\beta _i=1$$ for $$n \ge 1$$ and we get that

$$\begin{aligned} \det \varDelta _n=4(2-2q^2)^{n}(-4pq)^{ \sum _{k=0}^{n-1} (3^k-1)}(2-2p^2)^{n}(-4pq)^{\sum _{k=0}^{n-1} (3^k-1)} \end{aligned}$$

and thus

$$\begin{aligned} \det \varDelta _n=2^{2\cdot 3^n}(1-q^2)^{n}(1-p^2)^{n}(pq)^{(3^n-2n-1)} \end{aligned}$$

which gives us that

$$\begin{aligned} \log {\det \varDelta _n}=2\cdot 3^n \log {2}+n(\log {(1-q^2)}+\log {(1-p^2)})+(3^n-2n-1)\log {(pq)}. \end{aligned}$$

Since $\log {\det {\mathcal {L}}}=\log {pq}$ we obtain our result. $\square$

We can observe that for $p=q=\frac{1}{2}$ this corresponds to the standard combinatorial graph Laplacian on the cyclic graph $C_{2\cdot 3^n}$. Therefore (5.1) becomes

$$\begin{aligned} \log {\det \varDelta _n}=n\log {9}+\log {4} \end{aligned}$$

which is exactly as expected by observing that the cyclic graph has as many spanning trees as number of vertices and using Kirchhoff’s Matrix-Tree theorem. This is also equivalent to the formula

$$\begin{aligned} \prod _{k=1}^{2\cdot 3^n}\left( 2-2\cos {\frac{2k\pi }{2\cdot 3^n}}\right) =4\cdot 3^{2n}. \end{aligned}$$

## References

[1] Akkermans, E.: Statistical mechanics and quantum fields on fractals. In: Cafri, D., Lapidus, M.L., Pearse, E.P.J., van Frankenhuijsen M. (eds.) Fractal geometry and dynamical systems in pure and applied mathematics II: Fractals in applied mathematics. Contemporary Mathematics, vol. 601, pp. 1–21. American Mathematical Society, Providence, RI (2013)

[2] Akkermans, E., Dunne, G., Levy, E.: Wave propagation in one-dimension: methods and applications to complex and fractal structures. arXiv preprint arXiv:1210.7409 (2012)

[3] Akkermans E, Dunne GV, Teplyaev A (2009). Physical consequences of complex dimensions of fractals. EPL (Europhys. Lett.).

[4] Akkermans E, Dunne GV, Teplyaev A (2010). Thermodynamics of photons on fractals. Phys. Rev. Lett..

[5] Akkermans E, Gurevich E (2013). Spontaneous emission from a fractal vacuum. EPL (Europhys. Lett.).

[6] Anema JA, Tsougkas K (2016). Counting spanning trees on fractal graphs and their asymptotic complexity. J. Phys. A Math. Theor..

[7] Bajorin N, Chen T, Dagan A, Emmons C, Hussein M, Khalil M, Mody P, Steinhurst B, Teplyaev A (2008). Vibration modes of 3n-gaskets and other fractals. J. Phys. A Math. Theor..

[8] Chang S-C, Chen L-C, Yang W-S (2007). Spanning trees on the Sierpinski gasket. J. Stat. Phys..

[9] Chen JP, Molchanov S, Teplyaev A (2015). Spectral dimension and Bohr’s formula for Schrödinger operators on unbounded fractal spaces. J. Phys. A Math. Theor..

[10] Chen JP, Teplyaev A (2016). Singularly continuous spectrum of a self-similar Laplacian on the half-line. J. Math. Phys..

[11] Chinta G, Jorgenson J, Karlsson A (2010). Zeta functions, heat kernels, and spectral asymptotics on degenerating families of discrete tori. Nagoya Math. J..

[12] Derfel G, Grabner P, Vogl F (2008). The zeta function of the Laplacian on certain fractals. Trans. Am. Math. Soc..

[13] Derfel G, Grabner PJ, Vogl F (2012). Laplace operators on fractals and related functional equations. J. Phys. A Math. Theor..

[14] Dunne GV (2012). Heat kernels and zeta functions on fractals. J. Phys. A Math. Theor..

[15] Elizalde, E.: Ten physical applications of spectral zeta functions. Lecture Notes in Physics, 855, 2nd edn, pp. xiv+227. Springer, Heidelberg (2012). ISBN: 978-3-642-29404-4

[16] Elizalde E, Odintsov S, Romeo A, Bytsenko AA, Zerbini S (1994). Zeta Regularization Techniques with Applications.

[17] Englert F, Frère J-M, Rooman M, Spindel P (1987). Metric space-time as fixed point of the renormalization group equations on fractal structures. Nucl. Phys. B.

[18] Fuglede B, Kadison RV (1952). Determinant theory in finite factors. Ann. Math..

[19] Fukushima M, Shima T (1992). On a spectral analysis for the Sierpinski gasket. Potential Anal..

[20] Hawking SW (1977). Zeta function regularization of path integrals in curved spacetime. Commun. Math. Phys..

[21] Kigami J (2001). Analysis on Fractals.

[22] Kigami J, Lapidus ML (1993). Weyl’s problem for the spectral distribution of Laplacians on pcf self-similar fractals. Commun. Math. Phys..

[23] Knizhnik VG, Polyakov AM, Zamolodchikov AB (1988). Fractal structure of 2d—quantum gravity. Mod. Phys. Lett. A.

[24] Lapidus ML, Van Frankenhuysen M (2000). Fractal Geometry and Number Theory: Complex Dimensions of Fractal Strings and Zeros of Zeta Functions.

[25] Lauscher O, Reuter M (2005). Fractal spacetime structure in asymptotically safe gravity. J. High Energy Phys..

[26] Lyons R (2005). Asymptotic enumeration of spanning trees. Comb. Probab. Comput..

[27] Malozemov L, Teplyaev A (2003). Self-similarity, operators and dynamics. Math. Phys. Anal. Geom..

[28] Reuter M, Saueressig F (2011). Fractal space-times under the microscope: a renormalization group view on Monte Carlo data. J. High Energy Phys..

[29] Shima T (1991). On eigenvalue problems for the random walks on the Sierpinski pre-gaskets. Jpn. J. Ind. Appl. Math..

[30] Shima T (1996). On eigenvalue problems for Laplacians on pcf self-similar sets. Jpn. J. Ind. Appl. Math..

[31] Steinhurst BA, Teplyaev A (2013). Existence of a meromorphic extension of spectral zeta functions on fractals. Lett. Math. Phys..

[32] Strichartz R (2003). Fractafolds based on the Sierpinski gasket and their spectra. Trans. Am. Math. Soc..

[33] Strichartz R (2012). Exact spectral asymptotics on the Sierpinski gasket. Proc. Am. Math. Soc..

[34] Strichartz RS (2006). Differential Equations on Fractals: A Tutorial.

[35] Strichartz RS, Teplyaev A (2012). Spectral analysis on infinite Sierpiński fractafolds. J. d’Analyse Math..

[36] Tanese D, Gurevich E, Baboux F, Jacqmin T, Lemaître A, Galopin E, Sagnes I, Amo A, Bloch J, Akkermans E (2014). Fractal energy spectrum of a polariton gas in a Fibonacci quasiperiodic potential. Phys. Rev. Lett..

[37] Teplyaev, A.: Spectral zeta function of symmetric fractals. In: Bandt, C., Mosco, U., Zähle, M. (eds.) Fractal Geometry and Stochastics III, Progress in probability, vol. 57, pp. 245–262. Birkhäuser, Basel (2004)

[38] Teplyaev A (2007). Spectral zeta functions of fractals and the complex dynamics of polynomials. Trans. Am. Math. Soc..

[39] Teufl E, Wagner S (2011). The number of spanning trees in self-similar graphs. Ann. Comb..

[40] Vertman, B.: Regularized limit of determinants for discrete tori. ArXiv e-prints (2015)

